# Longitudinal effects of diet quality on healthy aging - Focus on cardiometabolic health: findings from the Canadian Longitudinal Study on Aging (CLSA)

**DOI:** 10.1007/s40520-025-03058-9

**Published:** 2025-05-16

**Authors:** Farhad Vahid, Piotr Wilk, Torsten Bohn

**Affiliations:** 1 Nutrition and Health Research Group, Department of Precision Health, Luxembourg Institute of Health, 1A, B Edison, Strassen, L-1445 Luxembourg; 2 Department of Epidemiology and Biostatistics, Western University, London, ON Canada

**Keywords:** Dietary indices, Vessel health, Hypertension prevention & control, Dietary patterns, Chronic disease, Nutritional biomarkers, CLSA

## Abstract

**Background:**

Hypertension, a major concern for older adults, contributes to morbidity and mortality by increasing the risk of cardiovascular disease, stroke, kidney dysfunction, and cognitive decline. A healthy diet plays a vital role in limiting chronic disease progression in aging populations.

**Aim:**

This study investigated the association between diet quality and healthy aging, focusing on blood pressure measurements (BPMs), using the Canadian Longitudinal Study on Aging (CLSA).

**Methods:**

Participants aged 45–85 years at baseline were followed for up to nine years. Mediterranean diet score (MDS) was determined based on the validated short diet questionnaire (SDQ). BP and mean arterial pressure (MAP) were measured at baseline (2010), follow-up 1 (2015), and follow-up 2 (2018). Linear regression models (LRMs), linear mixed-effects models (LMMs), and latent change score models (LCSMs) examined the associations and longitudinal effect between MDS and BPMs, adjusted for potential confounders. Individuals who participated in all three waves (*n* = 25,377) were included.

**Results:**

Fully adjusted LRMs showed significant (*p* < 0.001) inverse associations between MDS and all BPMs across all time points, e.g., 1 unit increase in the MDS (min0-max50) was associated with a 0.058 mmHg decrease of diastolic BP (DBP) (β=-0.058), 0.052 mmHg systolic BP (SBP) (β=-0.052), and 0.056 mmHg MAP (β=-0.056). LCSMs indicated that a 1-unit higher baseline MDS was significantly associated with 0.090 mmHg reductions in DBP at follow-up 2 (β=-0.090,*p* < 0.001), 0.078 mmHg for SBP (β=-0.078,*p* = 0.002) and 0.076 mmHg for MAP (β=-0.076,*p* = 0.003). Changes in MDS during follow-ups showed no consistent significant associations with BPMs at follow-up 1 or 2.

**Discussion and conclusion:**

Higher MDS was associated with lower BPMs over time. This study highlights the role of diet quality in healthy aging and mitigating cardiometabolic risk in older adults.

## Introduction

Healthy aging is an increasingly critical public health concern, driven by the rapid rise in the global elderly population [1]. According to projections by the United Nations, the global population aged ≥ 65 is expected to increase from 10% in 2022 to 16% by 2050 [2]. In developed regions, this demographic shift will be even more pronounced. For instance, Europe and North America are anticipated to see their populations aged ≥ 65 reach 27% by 2050 [2]. Concomitant with this demographic shift, there is an alarming surge in the prevalence of chronic conditions, deteriorated health, and reduced quality of life in older adults [3]. Noncommunicable diseases (NCDs), especially cardiovascular diseases (CVD), cancer, and type 2 diabetes (T2D), account for 74% of all deaths globally, with 41 million deaths annually due to these conditions [4, 5], with CVD contributing 17.9 million deaths per year as the leading cause of mortality [5]​. These numbers are expected to increase drastically in the following decades, in line with the increased proportion of the elderly population [6]. For example, between 2021 and 2050, the global age-standardized total diabetes prevalence is expected to increase by 59.7%, from 6.1 to 9.8%, resulting in 1.31 billion people living with diabetes in 2050 [7]. In addition, by 2050, global ischemic heart disease (strongly related to hypertension) incidence, prevalence, deaths, and disability adjusted life-years (DALYs) are projected to reach 67.3 million, 510 million, 16 million, and 302 million, respectively, which represents an increase of 116%, 106%, 80%, and 62% from 2021 [8] and hypertension by 20–30% by 2050 [9, 10]. In many countries, older adults are at heightened risk of developing these diseases due to a combination of aging, non-optimal lifestyle patterns, and limited access to healthcare [11]. Data from the European Health Examination Survey (EHES) conducted in Luxembourg, for instance, reveal that nearly 31% of the population aged 15 and over exhibit elevated blood pressure (BP), and over 70% of those were either unaware of their condition or not adequately controlled, and with an even higher proportion being older adults [12]. These high numbers pose considerable challenges for healthcare systems, as the growing burden of associated CVD results in increased multimorbidity, mortality, and healthcare utilization and needs [3].

An essential factor of CVD is vessel health, with BP as the most prominent marker indicating vessel stiffness and damage, increasing the risk of stroke [13] and heart attacks [14]. Regarding elevated BP, the interplay between biological (such as age), environmental (such as socio-economic aspects), and behavioral factors (such as diet and physical inactivity) are major challenges in managing this chronic condition [15]. Limited physical activity (PA) [16], excess pollution such as by chemical contaminants [17], light and noise [18], and unhealthy dietary patterns such as consumption of food items rich in salt [19], as present in processed foods, are regarded as the main drivers for elevated BP. Research has highlighted overall diet quality as a pivotal factor for preventing elevated BP. Specifically, dietary patterns that emphasize nutrient-dense foods, low presence of processed foods, and sufficient amounts of dietary fiber but low in salt and simple sugars, such as the Mediterranean diet (MD), have been linked to a reduced risk of cardiometabolic diseases, including hypertension [20, 21], T2D [22], and CVD [23].

On the other hand, although the aging process has long been understood to involve complex, multifaceted changes throughout an individual’s life, the cumulative effects of these changes remain inadequately defined. Aging is influenced by a dynamic interplay of biological, psychological, and sociodemographic factors, all of which are subject to change over time. These factors, combined with dietary patterns (generally referring to the overall types of foods and beverages consumed regularly over time) and eating habits (the specific behaviors or routines related to eating, such as meal frequency, timing, and portion size), profoundly affect health outcomes as they accumulate over the lifespan [24]. The effects of dietary patterns/habits on aging have been reported to be particularly significant, as they can enormously exacerbate the progression of age-related diseases, ultimately influencing longevity and health span [25, 26]. Understanding how these factors interact with age-related biological changes over time could be vital to addressing chronic disease risk and promoting healthier aging trajectories globally.

This study sought to investigate the longitudinal effect and cross-sectional association between diet quality and healthy aging, specifically focusing on BP, using Canadian Longitudinal Study on Aging (CLSA) data. By examining blood pressure measures (BPMs), including mean arterial pressure (MAP) over time, this research aims to provide insights into how following dietary patterns contribute to health outcomes in later life. We hypothesized that higher diet quality, particularly adherence to the MD over extended periods of time, would be associated with better cardiovascular health outcomes, i.e., lower BP, in older adults. Findings could have important implications for dietary guidelines and public health interventions to improve quality of life and reduce chronic disease prevalence among older adults.

## Methods and materials

### Study design and population

The Canadian Longitudinal Study on Aging (CLSA) is a large, national, prospective cohort study designed to explore health, aging, and quality of life over time among Canadians aged 45 to 85 years at baseline [27, 28]. Initiated in 2009, the CLSA aims to follow approximately 50,000 participants for a period of at least 20 years, with regular data collection at baseline and follow-up intervals every three years. In addition, CLSA enhances its research capabilities through data linkages with various administrative health databases.

The inclusion criteria required participants to fall within the 45–85 age range at baseline, be able to respond in either English or French and have the capacity to provide informed consent. Exclusion criteria comprised individuals residing in Canada’s three territories (Yukon, Northwest Territories, and Nunavut), those living on federal First Nations reserves or other First Nations settlements, full-time members of the Canadian Armed Forces, residents of long-term care institutions providing 24-hour nursing care, and individuals with cognitive impairments at baseline that precluded their ability to provide informed consent were excluded at recruitment. These criteria were established to ensure a representative and capable cohort for longitudinal assessment [27, 28].

At recruitment, approximately 90% of participants consented to provide their health insurance numbers, facilitating the linkage of CLSA data with existing health records. These linkages aimed to gather comprehensive information on medication usage, health service utilization, and hospital and physician visits and ascertain mortality data, including causes of death [27, 28]. Furthermore, the CLSA collaborates with the Health Data Research Network (HDRN) Canada to integrate data from provincial health care registries. This collaboration offers researchers access to enriched datasets, enabling more in-depth studies on aging and health outcomes [27, 28]. The project utilizes a stratified sampling method to ensure representation across age groups, sex, and regions within Canada.

**Fig. 1:**
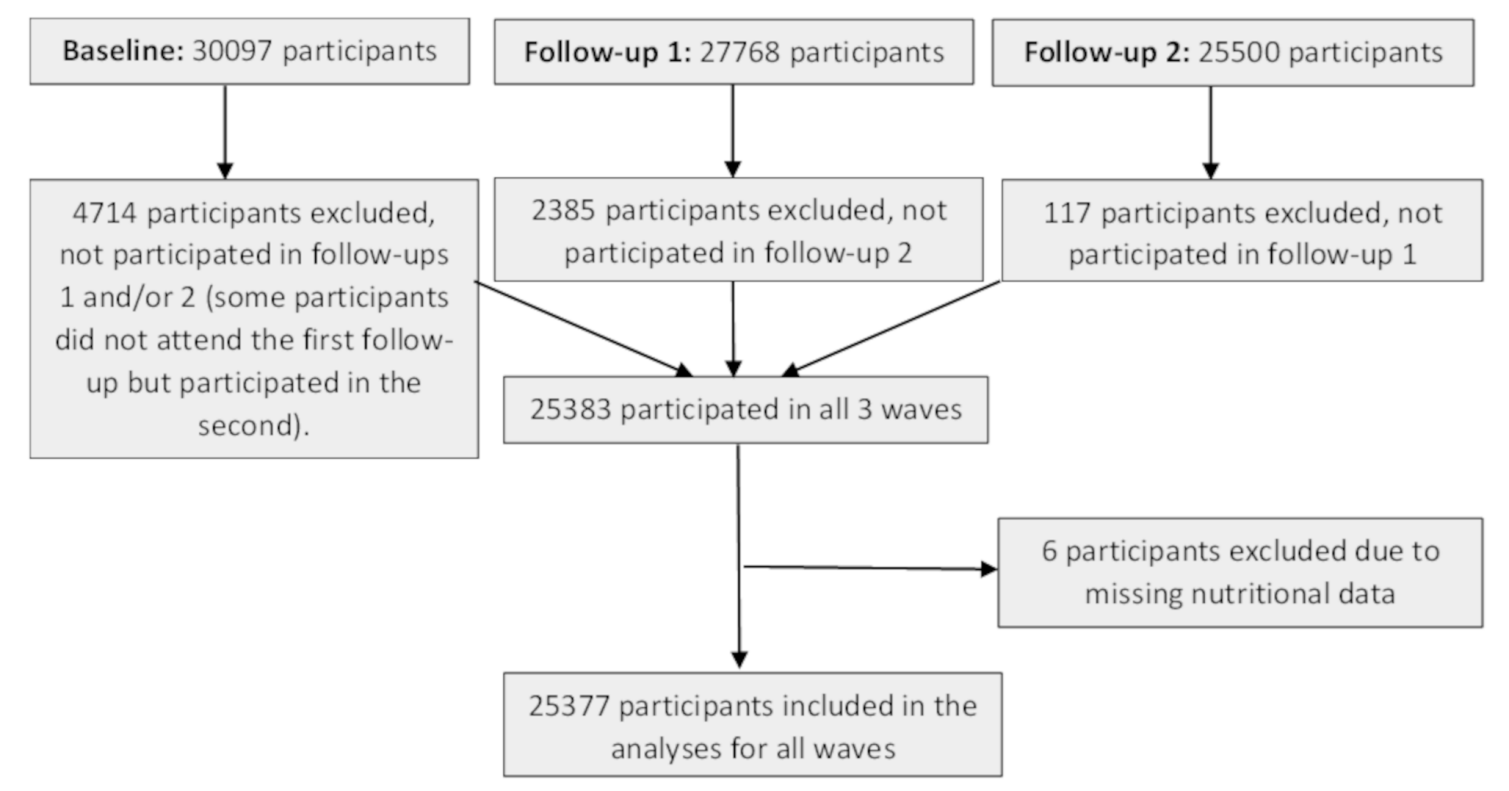
Participant Flow Diagram

In this article, data from the comprehensive cohorts at baseline (*n* = 30097; 2010), follow-up 1 (*n* = 27768; 2015), and follow-up 2 (*n* = 25500; 2018) were utilized. However, only individuals who participated in all three waves (*n* = 25383) were included in the analyses. Six participants were excluded due to missing nutritional data, and finally, 25,377 participants were included in the analyses for all waves (Fig. 1). We included only those participants who had BP data from all three waves (baseline, follow-up 1, and follow-up 2), ensuring that each participant contributed at least three BP measurement time points to the longitudinal models. Thus, no participants with only one BP measurement were included in the current study.

### Data collection and measurements

Data in the CLSA was collected through two primary modules: the comprehensive cohorts and the tracking cohorts. The comprehensive cohorts involved in-depth, in-person assessments at designated data collection sites across Canada, while the tracking cohorts relied on telephone interviews to gather data from a geographically dispersed sample. Data collected included information on sociodemographic characteristics, health behaviors, physical measures, chronic conditions, and functional status.

### Health and lifestyle data

Participants provided data on diet, PA, tobacco and alcohol use, sleep patterns, and other lifestyle factors. Nutritional assessment was conducted using a short diet questionnaire (SDQ), a validated tool designed to provide an overview of an individual’s dietary habits [29]. The SDQ includes a set of concise questions that capture the frequency and quantity of consumption of various food groups, allowing for an evaluation of overall dietary patterns [29]. This assessment method is particularly useful for large-scale studies where detailed dietary tracking may not be feasible. The SDQ was administered at multiple time points (e.g., baseline, follow-ups 1 and 2) to ensure a comprehensive evaluation of dietary habits over the study period. Additionally, the tool identified specific dietary factors, such as nutrient intake and food variety, which are essential for understanding their association with health outcomes. By employing the SDQ, the CLSA aimed to accurately assess the dietary intake with respect to selected food groups, e.g., cereals, fruits, vegetables, legumes, nuts, potatoes, fish, meat and meat products, poultry, and dairy products, among others, of participants, while minimizing the burden on respondents, ensuring reliable data for the analysis of diet-disease associations. Based on the data extracted from the SDQ, diet quality was assessed using the Mediterranean diet score (MDS), which was calculated based on food intake patterns at each wave [30]. The MDS ranged from a score from 0 to 50 and included elements on the intake of cereals, fruits, vegetables, legumes, nuts, potatoes, and fish as beneficial components and meat and meat products, poultry, full-fat dairy products, and alcohol as detrimental components (Table 1).

**Table 1:** Scoring algorithms for Mediterranean diet score (MDS)

**Components**	**Effect on the score**
1. cereals, non-refined (whole bread, pasta, rice, other grain, biscuits, etc.)	+
2. fruits	+
3. vegetables	+
4. legumes and nuts	+
5. potatoes	+
6. fish	+
7. meat and meat products	-
8. poultry	-
9. full-fat dairy products (like cheese, yogurt, and milk)	-
10. alcohol*	-
**Total (range)**	0–50

Using sex-specific quartile cut-offs, a score ranging from 0 to 5 was assigned to each of the ten components of the Mediterranean Diet. For beneficial components, such as vegetables, legumes and nuts, fruits, cereals, potatoes, and fish, participants who reported no consumption received a score of 0, while higher intake corresponded to higher quartile scores, up to a maximum of 5 points. For components considered detrimental, such as meat and meat products, poultry, full-fat dairy products (e.g., cheese, yogurt, and milk), and alcohol, the scoring was reversed, with higher intakes receiving fewer points. Consequently, the total MDS ranged from 0, indicating minimal adherence, to 50, reflecting maximal adherence to the Mediterranean Diet

* < 300 mL/d: 5 points, and > 700 or 0 mL/d: 0 points

### Cardiometabolic health outcomes

Cardiometabolic health markers, including BPMs, were measured across waves to track participant health changes over time. BPMs, the primary outcomes in this study, were measured six times per visit by trained personnel to ensure higher accuracy and reliability, with the average reading recorded for analysis. This approach aided in mitigating variability caused by factors such as stress, body position, and other momentary influences on BP. Additionally, we calculated the mean arterial pressure (MAP) using the following formula: MAP = DPB + 1/3(SBP– DBP) [31], where DBP represents diastolic BP, and SBP represents systolic BP. The MAP (with a range of 70 to 100 mmHg considered to be optimal for cardiovascular health [32]) provides a more comprehensive measure of the overall BP load on the circulatory system, accounting for the time spent in the diastole versus systole during the cardiac cycle [31, 33]. This calculation is instrumental in assessing cardiovascular risk, as it has been closely associated with vital organ functions, including the brain, kidneys, and heart [31, 33]. By incorporating MAP into our study, we aimed to capture a more holistic picture of participants’ cardiovascular health and its potential association with diet and aging.

## Statistical analyses

### Descriptive statistics and linear regression models

We assessed normality using visual inspection of Q-Q plots and checked homoscedasticity through residual plots, with no significant violations of assumptions detected. Descriptive statistics were calculated for all relevant variables, including means, standard deviations (SD), and frequencies (n, %). Cross-sectional associations between the MDS (as the dependent variable) and cardiometabolic health outcomes, including DBP, SBP, and MAP (as independent variables), were evaluated using linear regression models (LRMs). For each outcome, three models were employed, with confounders chosen following literature review: Model A was the crude model, assessing the unadjusted association between MDS and the respective BPMs; Model B was adjusted for age and sex, accounting for potential confounding by these demographic factors; Model C was further adjusted for marital status, dwelling status (own, renting, other), smoking status (current frequency cigarettes smoked), the total time to complete the 4-meter walk test (in seconds), and body mass index (BMI), to control for a broader range of potential confounders.

### Linear mixed models (LMM)

In addition to cross-sectional analyses, longitudinal data were analyzed using LMMs to account for within-subject associations across the three measurement waves. LMMs allowed for examining changes in BPMs over time as a function of the MDS while adjusting for time-invariant and time-varying covariates. By incorporating random effects, these models accounted for individual variability at baseline levels and trajectories over time, providing a more robust longitudinal data analysis within repeated measures.

The LMMs were specified as follows:

- Fixed effects: models were adjusted for age and sex, with MDS and its interactions with sex and waves as predictors to capture potential time-by-diet and sex-by-diet interactions;
- Random effects: random intercepts and slopes for waves and MDS were specified for each participant (SUBJECT = ID), allowing the model to estimate variability in baseline BPMs and change over time at the individual level. This specification captured the individual differences in BPMs trajectories associated with MDS;
- Covariance structure: an unstructured covariance structure (COVTYPE = UN) was used to model the repeated measures over time, allowing for flexible estimation of associations between time points within individuals.

The SPSS syntax used for these analyses, utilizing the REML (Restricted Maximum Likelihood) estimation method, specified the model to include fixed effects for main predictors and their interactions, while random effects were specified for waves and MDS within each participant. Least significant difference (LSD) adjusted pairwise comparisons of BPMs levels across waves were also generated, providing insights into temporal trends and changes over time. Additionally, the estimated marginal means (EMMEANS) of BPMs were calculated overall and across waves, allowing a straightforward interpretation of average effects within each measurement wave. Fixed predictors from the LMM were saved for further evaluation and visualization of model results. This approach enabled us to evaluate how dietary quality, measured by MDS, impacted BPMs longitudinally, accounting for within-subject variability and adjusting for key demographic and lifestyle covariates.

### Latent change score models

**Fig. 2:**
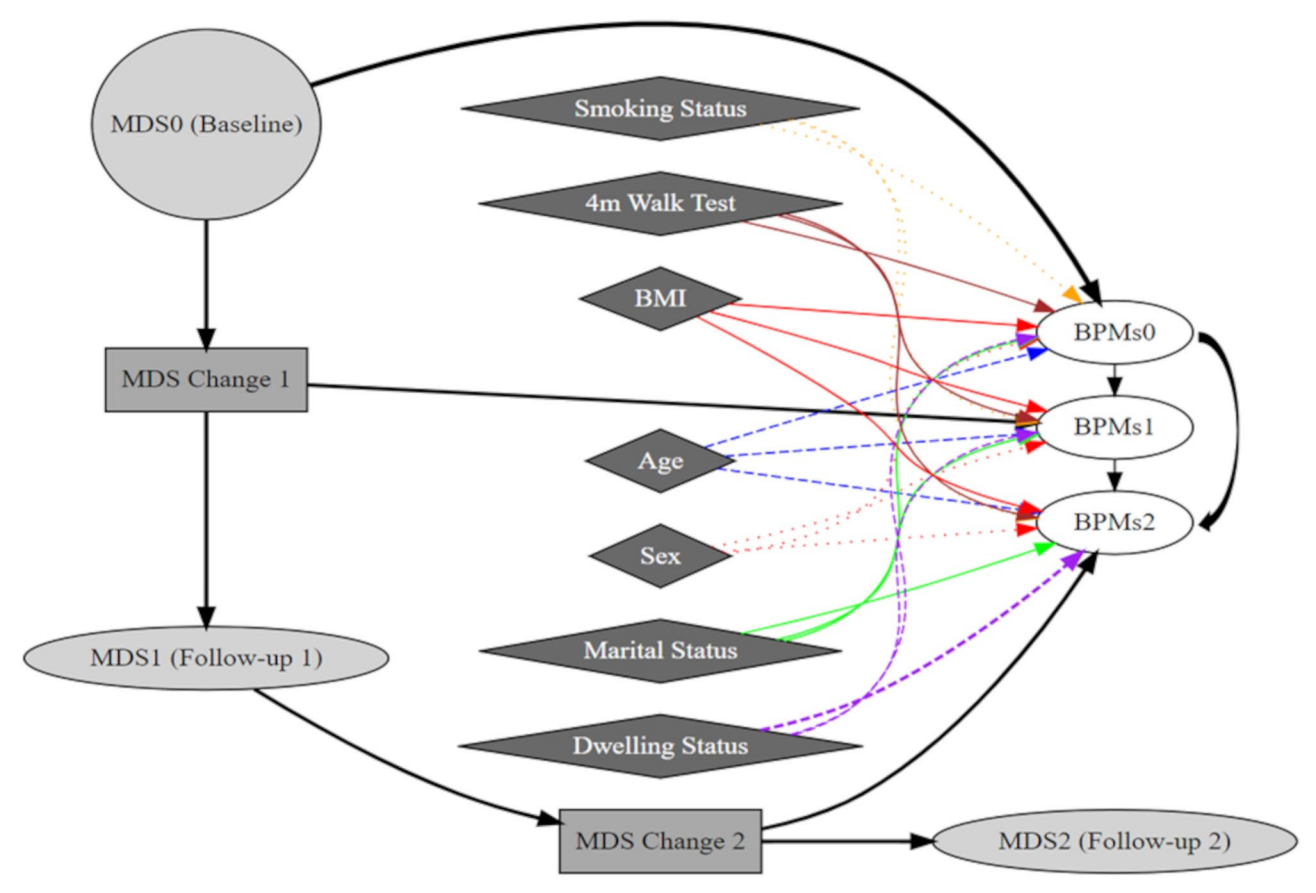
Latent change score diagram for the longitudinal impact of Mediterranean Diet Score (MDS) on blood pressure measurements (BPMs), adjusted for covariates

In addition, latent change score models (LCSM) were employed to investigate outcome changes over time, specifically focusing on the changes in MDS and BPMs between the baseline and the two follow-ups. These models allowed for estimating latent change scores (LCS), which captured the differences in MDS and BPMs between consecutive measurement points (Fig. 2).

In the present study, we used the Lavaan package in R to define and estimate the LCSM. The model was specified with latent change factors for MDS, where the change in MDS between baseline (MDS0), follow-up 1 (MDS1), and follow-up 2 (MDS2) was modeled. Specifically:

- MDS_change1 and MDS_change2 were defined as latent factors representing the change in MDS from baseline to follow-up 1 and from follow-up 1 to 2, respectively.
- The associations between MDS at each time point were modeled as follows:
  - MDS1 ~ 1*MDS0 (change in MDS from baseline to follow-up 1).
  - MDS2 ~ 1*MDS1 (change in MDS from follow-up 1 to follow-up 2).

The model further explored the impact of changes in MDS on BPMs, e.g., MAP, and adjusting for age and sex, among others, at each time point. The BPMs at each wave were regressed on the corresponding MDS (Model A: crude models), as well as on age and sex (Model B: adjusted for age and sex) and also Model C (full model: adjusted for age, sex, marital status, dwelling status, smoking status (current frequency cigarettes smoked), total time to complete the 4-meter walk test (in seconds), and BMI. As an example, Model B was:

- MAP0 ~ MDS0 + age0 + sex (Baseline MAP regressed on baseline MDS, age, and sex).
- MAP1 ~ MDS_change1 + age1 + sex (Follow-up 1 MAP regressed on change in MDS and adjustments for age and sex).
- MAP2 ~ MDS_change2 + age2 + sex (Follow-up 2 MAP regressed on change in MDS and adjustments for age and sex).

The model also included the measurement of indirect effects, where changes in MDS at earlier waves (e.g., MDS_change1) influenced later MDS (e.g., MDS_change2). This approach provided insight into the longitudinal effects of dietary changes, particularly MDS, on BPMs changes over time while controlling for demographic factors.

In summary, three models were used in LCSM, similar to the LRMs: Model A, Model B, and Model C (see above). These models examined how dietary changes (MDS) over time impacted cardiometabolic outcomes (DBP, SBP, MAP) while adjusting for relevant covariates.

Model fit was assessed using standardized estimates, including the Comparative Fit Index (CFI), Root Mean Square Error of Approximation (RMSEA), and Standardized Root Mean Square Residual (SRMR). Missing values in the data were handled using maximum likelihood estimation (ML) within the Lavaan package. Statistical significance was set at a *p*-value of < 0.05 (2-sided). Statistical analyses were conducted using IBM SPSS (version 25) and RStudio (version 4.4.1).

## Results

Characteristics of the study participants (*n* = 25377), including demographic, clinical, and lifestyle factors at the baseline, follow-up 1, and follow-up 2, are presented in Table 2. BMI remained relatively stable across the waves, while waist and hip circumference, waist-to-hip ratio, and BPMs (DBP, SBP, MAP) showed minimal variation over time. The 4 m walk times decreased slightly, reflecting changes in physical function. The MDS (min 0– max 49) declined from 29.8 ± 5.3 at baseline to 29.0 ± 5.4 at follow-up 1 and 28.3 ± 5.6 at follow-up 2. Demographic variables such as marital status, dwelling status, and sex remained consistent, with a slight increase in the number of widowed participants and those living alone.

**Table 2:** Characteristics of study participants: demographics, clinical profiles, and lifestyle factors (*n* = 25377)

Variables	Baseline			Follow-up 1			Follow-up 2		
	Mean ± SD	Min-Max	missing	Mean ± SD	Min-Max	missing	Mean ± SD	Min-Max	missing
**Age (years)**	62.1 ± 9.8	45–86	0	65.1 ± 9.8	46–89	0	67.9 ± 9.8	49–93	0
**BMI (kg/m**^**2**^)	28.0 ± 5.4	12.9–69.6	79	28.0 ± 5.5	12.1–72.6	238	27.7 ± 5.5	12.8–90.9	631
**Hip circumference (cm)**	104.1 ± 11.5	50.0-190.0	146	102.8 ± 11.8	63.5-183.5	812	101.8 ± 11.7	68.4–183.0	11,269
**Waist-to-hip ratio**	0.90 ± 0.09	0.50–1.81	146	0.91 ± 0.10	0.47–1.55	812	0.91 ± 0.09	0.45–1.91	11,269
**Waist circumference (cm)**	93.9 ± 14.5	53.0-180.0	146	93.9 ± 14.7	54.0-179.0	812	93.0 ± 14.5	54.0-194.5	11,269
**DBP (mmHg)**	74.4 ± 9.8	44.6–151.0	210	73.1 ± 9.8	40.0-139.5	875	73.1 ± 9.8	42.0-159.0	11,343
**Pulse (mmHg)**	71.3 ± 11.4	38.1-150.5	210	71.3 ± 11.6	37.0-162.8	875	71.3 ± 11.5	38.7-123.2	11,343
**SBP (mmHg)**	121.6 ± 16.5	72.3–234.0	210	121.3 ± 16.5	68.0-233.0	875	122.2 ± 16.7	71.2-216.7	11,343
**MAP (mmHg)**	90.2 ± 11.0	55.2–177	210	89.1 ± 10.8	52.4-162.3	875	89.5 ± 10.8	53.1-163.7	11,343
**Average grip strength (kg)**	33.8 ± 11.4	0.2–84.1	1802	32.9 ± 11.3	1.2–79.1	1820	31.6 ± 11.0	6.1–78.1	11,695
**Total time required to complete 4 mWalk (in seconds)**	4.2 ± 1.0	1.5–37.8	235	4.3 ± 1.1	2.0-45.9	1419	4.4 ± 1.1	1.3–20.3	11,421
**MDS**	29.8 ± 5.3	0–49	0	29.0 ± 5.4	0–49	0	28.3 ± 5.6	0–49	0
**Variables**	**n (%)**			**n (%)**			**n (%)**		
**Sex**	25,377 (100)			-			-		
- Women	12,955 (51.2)			-			-		
- Men	12,382 (48.8)			-			-		
**Marital status**	25,369 (99.9)			25,367 (99.9)			25,365 (99.9)		
- Single, never married	2162 (8.5)			2267 (8.9)			2146 (8.5)		
- Married, living with partner	17,881 (70.5)			17,493 (68.9)			17,092 (67.4)		
- Widowed	2082 (8.2)			2395 (9.4)			2844 (11.2)		
- Divorced	2600 (10.2)			2507 (9.9)			2560 (10.1)		
- Separated	644 (2.5)			705 (2.8)			723 (2.8)		
**Dwelling status**	25,357 (99.9)			25,358 (99.9)			25,331 (99.8)		
- Own	21,846 (86.1)			21,351 (84.1)			20,795 (81.9)		
- Rent	3346 (13.2)			3703 (14.6)			4190 (16.5)		
- Other	165 (0.7)			304 (1.2)			436 (1.4)		
**Smoking status**	1920 (7.6)			1669 (6.6)			1439 (5.7)		
- Daily	1512 (6.0)			1341 (5.3)			1211 (4.8)		
- Occasionally	408 (1.6)			328 (1.3)			228 (0.9)		
Current number of cigarettes smoked per day*	1512 (6.0)			1341 (5.3)			1209 (4.8)		
- 1–5 cigarettes	205 (0.8)			206 (0.8)			202 (0.8)		
- 6–10 cigarettes	381 (1.5)			344 (1.4)			337 (1.3)		
- 11–15 cigarettes	353 (1.4)			311 (1.2)			269 (1.1)		
- 16–20 cigarettes	293 (1.2)			230 (0.9)			188 (0.7)		
- 21–25 cigarettes	204 (0.8)			190 (0.7)			162 (0.6)		
- 26 + cigarettes	76 (0.3)			60 (0.2)			51 (0.2)		
**Taking medication for hypertension**	8787 (34.6)			9376 (36.9)			9935 (39.1)		
- Yes	6945 (27.4)			7635 (30.1)			8273 (32.6)		
- No	1842 (7.3)			1741 (6.9)			1662 (6.5)		

* Only smokers

BMI: Body Mass Index, DBP: Diastolic Blood Pressure, SBP: Systolic Blood Pressure, MAP: Mean Arterial Pressure, MDS: Mediterranean Diet Score

Results from LRMs examining the association between diet quality, measured by the MDS, and BPMs (DBP, SBP, and MAP) at baseline and follow-ups are presented in Table 3. Model A (crude model) revealed significant associations between MDS and all BPMs across all time points (*p* < 0.001), with a slight reduction in effect size at follow-up 2 for DBP and MAP. Model B (adjusted for age and sex) demonstrated similar trends, with slightly attenuated associations compared to Model A. Model C (fully adjusted for additional covariates) showed weaker associations but is still significant for DBP, SBP, and MAP at all follow-ups. The results highlight a consistent inverse association between MDS and BPMs.

**Table 3:** Linear regression models: association between diet quality (MDS) and blood pressure measurements in each follow-up

Models	Dependent variables**	Baseline				Follow-up 1				Follow-up 2*			
		Unstandardized β (95%CI)	Standardized β	*R* ^2^	*p*-value	Unstandardized β (95%CI)	Standardized β	*R* ^2^	*p*-value	Unstandardized β (95%CI)	Standardized β	*R* ^2^	*p*-value
Model A	DBP (mmHg)	−0.20 (− 0.23, − 0.18)	−0.11	0.014	**< 0.001**	−0.18 (− 0.21, − 0.16)	−0.10	0.010	**< 0.001**	0.19 (− 0.22, − 0.17)	−0.11	0.012	**< 0.001**
	SBP (mmHg)	−0.10 (− 0.14, − 0.06)	−0.03	0.001	**< 0.001**	−0.08 (− 0.12, − 0.04)	−0.03	0.001	**< 0.001**	−0.10 (− 0.15, − 0.05)	−0.03	0.001	**< 0.001**
	MAP (mmHg)	−0.17 (− 0.19, − 0.14)	−0.08	0.007	**< 0.001**	−0.15 (− 0.17, − 0.12)	−0.07	0.006	**< 0.001**	−0.16 (− 0.19, − 0.13)	−0.08	0.007	**< 0.001**
Model B*	DBP (mmHg)	−0.11 (− 0.13, − 0.08)	−0.06	0.085	**< 0.001**	−0.09 (− 0.11, − 0.07)	−0.05	0.092	**< 0.001**	−0.11 (− 0.14, − 0.08)	−0.06	0.089	**< 0.001**
	SBP (mmHg)	−0.19 (− 0.23, − 0.15)	−0.06	0.087	**< 0.001**	−0.17 (− 0.20, − 0.13)	−0.05	0.067	**< 0.001**	−0.19 (− 0.24, − 0.14)	−0.06	0.056	**< 0.001**
	MAP (mmHg)	−0.13 (− 0.16, − 0.11)	−0.06	0.041	**< 0.001**	−0.12 (− 0.14, − 0.09)	−0.06	0.029	**< 0.001**	−0.14 (− 0.17, − 0.10)	−0.07	0.020	**< 0.001**
Model C*	DBP (mmHg)	−0.05 (− 0.07, − 0.02)	−0.02	0.126	**0.001**	−0.04 (− 0.06, − 0.01)	−0.02	0.134	**0.002**	−0.06 (− 0.09, − 0.03)	−0.03	0.126	**< 0.001**
	SBP (mmHg)	−0.10 (− 0.14, − 0.05)	−0.03	0.155	**< 0.001**	−0.05 (− 0.09, − 0.02)	−0.02	0.149	**0.006**	−0.09 (− 0.13, − 0.04)	−0.03	0.131	**0.001**
	MAP (mmHg)	−0.06 (− 0.09, − 0.03)	−0.03	0.104	**< 0.001**	−0.04 (− 0.06, − 0.02)	−0.02	0.101	**0.001**	−0.07 (− 0.10, − 0.04)	−0.03	0.084	**< 0.001**

Model A: crude model, without any adjustment,

Model B: adjusted for age and sex,

Model C: Model B + adjusted for marital status, dwelling status, smoking status (current frequency of cigarettes smoked), total time to complete the 4-meter walk test (in seconds), and body mass index (BMI)

DBP: Diastolic Blood Pressure, SBP: Systolic Blood Pressure, MAP: Mean Arterial Pressure,

**The Mediterranean Diet Score (MDS) is considered the independent variable in all models

* Adjusted R^2^ are reported

The LMMs results for BPMs (DBP, SBP, and MAP) across three time points (baseline, follow-up 1, follow-up 2), adjusted for age and sex, are presented in Table 4. DBP significantly decreased between baseline and follow-up 1 (0.83 mmHg, *p* < 0.001) and from follow-up 1 to follow-up 2 (0.23 mmHg, *p* = 0.020). SBP showed a significant reduction at both follow-ups compared to baseline (1.7 mmHg, *p* < 0.001; 2.1 mmHg, *p* < 0.001), with a minor change between follow-up 1 and follow-up 2 (0.38 mmHg, *p* = 0.027). MAP decreased at both follow-ups compared to baseline (1.1 mmHg, *p* < 0.001; 0.85 mmHg, *p* < 0.001), with a slight increase from follow-up 1 to follow-up 2 (-0.27 mmHg, *p* = 0.018).

**Table 4:** Results from linear mixed model analysis of blood pressure measurements by MDS controlling for age and sex

	Estimates (95%CI)			Pairwise comparisons					
	Baseline	FUP1^b^	FUP2^c^	Mean Difference ^a−b^	*p*-value ^a−b^	Mean Difference ^a−c^	*p*-value ^a−c^	Mean Difference ^b−c^	*p*-value ^b−c^
DBP (mmHg)	74.0 (73.9–74.1)	73.2 (73.0-73.3)	73.8 (73.6–73.9)	0.83	**< 0.001**	0.23	**0.020**	0.60	**< 0.001**
SBP (mmHg)	122.7 (122.5-122.8)	121.0 (120.7-121.1)	120.5 (120.3-120.8)	1.70	**< 0.001**	2.10	**< 0.001**	0.38	**0.027**
MAP (mmHg)	90.2 (90.1–90.4)	89.1 (88.9–89.2)	89.4 (89.1–89.5)	1.10	**< 0.001**	0.85	**< 0.001**	-0.27	**0.018**

MDS: Mediterranean Diet Score, DBP: Diastolic Blood Pressure, SBP: Systolic Blood Pressure, MAP: Mean Arterial Pressure, FUP1: Follow-up 1, FUP2: Follow-up 2

Table 5 presents the results of the LCSMs examining BPMs (DBP, SBP, and MAP) in association with the MDS across three time points. For all models (A, B, and C), significant inverse associations between baseline MDS (MDS0) and BPMs were observed, with values ranging from − 0.031 to -0.090 (*p* < 0.001). At follow-up 2, significant inverse associations were observed between MDS change and DBP, SBP, and MAP across all models, with *p*-values ranging from 0.001 to 0.011 (Table 5). In contrast, changes in MDS during follow-ups (MDS_change1 and MDS_change2) did not exhibit significant associations with BPMs at follow-up 1, and the results at follow-up 2 were mixed. The model fit indices (RMSEA, SRMR, CFI) were within acceptable ranges, indicating a good fit for all three models (Table 5).

**Table 5:** Latent change score model results for BPMs by MDS

Models	Measurements	MDS0 (β) on BPMs0	*p*-value	MDS_change1 (β) on BPMs1	*p*-value	MDS_change2 (β) on BPMs2	*p*-value	RMSEA (95%CI)	SRMR	CFI
Model A	DBP (mmHg)	-0.075	*p* < 0.001	0.006	*p* = 0.515	-0.040	*p* = 0.001	0.116 (0.113–0.120)	0.062	0.953
	SBP (mmHg)	-0.057	*p* < 0.001	-0.008	*p* = 0.648	-0.038	*p* = 0.087	0.108 (0.104–0.112)	0.036	0.957
	MAP (mmHg)	-0.072	*p* < 0.001	0.002	*p* = 0.849	-0.040	*p* = 0.006	0.112 (0.108–0.116)	0.050	0.953
Model B	DBP (mmHg)	-0.053	*p* < 0.001	-0.004	*p* = 0.668	-0.040	*p* = 0.001	0.066 (0.064–0.068)	0.034	0.957
	SBP (mmHg)	-0.090	*p* < 0.001	0.001	*p* = 0.993	-0.036	*p* = 0.097	0.066 (0.064–0.069)	0.032	0.954
	MAP (mmHg)	-0.065	*p* < 0.001	-0.002	*p* = 0.831	-0.039	*p* = 0.007	0.066 (0.064–0.068)	0.034	0.952
Model C	DBP (mmHg)	-0.031	*p* < 0.001	-0.005	*p* = 0.572	-0.038	*p* = 0.002	0.040 (0.038–0.041)	0.022	0.944
	SBP (mmHg)	-0.042	*p* = 0.004	-0.001	*p* = 0.930	-0.032	*p* = 0.137	0.038 (0.037–0.039)	0.020	0.946
	MAP (mmHg)	-0.035	*p* < 0.001	-0.004	*p* = 0.711	-0.036	*p* = 0.011	0.039 (0.038–0.040)	0.021	0.941

Model A: crude model, without any adjustment,

Model B: adjusted for age and sex,

Model C: Model B + adjusted for marital status, dwelling status, smoking status (current frequency of cigarettes smoked), total time to complete the 4-meter walk test (in seconds), and body mass index (BMI)

CFI: Comparative Fit Index, RMSEA: Root Mean Square Error of Approximation, SRMR: Standardized Root Mean Square Residual, MDS: Mediterranean Diet Score, DBP: Diastolic blood pressure, SBP: Systolic blood pressure, MAP: Mean arterial pressure, BPMs: Blood pressure measurements

## Discussion

This study investigated the associations and effects of healthy dietary patterns on BP in the older adults of the Canadian population. Our findings demonstrate a significant association between higher adherence to the MDS and improved BP as a marker of CVD. In fully adjusted models, a one-unit increase in MDS (out of a maximum scale of 50) was associated with reductions of 0.058 mmHg in DBP, 0.052 mmHg in SBP, and 0.056 mmHg in MAP. Longitudinal analyses further highlighted the sustained benefits of, e.g., one unit higher baseline MDS on BP outcomes at follow-up 2, with reductions of 0.090 mmHg for DBP, 0.078 mmHg for SBP, and 0.076 mmHg for MAP. Changes in MDS over time showed mixed associations with BP at later follow-ups, suggesting the importance of consistent dietary adherence. Although the effect sizes observed in this study are modest, even small reductions in BPMs over time can lead to meaningful long-term health benefits, particularly in aging populations at increased risk for CVD. These findings highlight the importance of promoting sustainable dietary changes to improve cardiovascular health in older adults, even when the individual effects may seem subtle. Overall, our results align with previous evidence that greater adherence to a Mediterranean-style diet positively influences BP and underscores the diet’s potential role in promoting cardiometabolic health in aging populations.

Our findings are consistent with previous research demonstrating the cardioprotective effects of adherence to the MD, which is rich in fruits, vegetables, and healthy fats, as well as being low in sugar and processed foods [34–36]. Numerous studies have established an inverse association between diet quality, particularly high adherence to the MD, and BP levels [37, 38]. For instance, a meta-analysis of controlled trials in adults showed that individuals with higher MDS exhibited significantly (*p* < 0.001) lower SBP (mean difference (95%CI): −1.34 (− 2.00, − 0.67)) and DBP (mean difference (95%CI): −0.81 (− 1.30, − 0.32)) mmHg [39]. However, they concluded that pooled findings should be interpreted cautiously due to the substantial between-study heterogeneity found for most analyses and the small number of studies for some outcomes [39].

Similarly, longitudinal studies, such as the PREDIMED randomized trial, have highlighted the potential of the MD to reduce hypertension risk and improve overall cardiometabolic health outcomes [40]. The PREDIMED trial reported that the percentage of participants with healthy BP increased in the intervention groups with MD (*p*-value for within-group changes < 0.001). After 4 years of follow-up, participants in the MD groups had significantly lower DBP than the participants in the control group (− 1.53 mmHg (95%CI: −2.01 to − 1.04). However, they reported no between-group differences in changes in SBP [40].

Our study extends these findings by employing a comprehensive analytical approach, including linear mixed and latent change score models, to explore cross-sectional and longitudinal associations. Unlike many prior studies, our research captures the dynamic association between diet quality and changes in BP over nearly a decade, providing robust evidence for the long-term benefits of maintaining a high-quality diet in older adults. Furthermore, while most studies have focused on SBP and DBP separately, our inclusion of MAP as a composite marker adds nuance and robustness to understanding how dietary patterns influence vascular health.

Another critical aspect of our findings is the differential impact of baseline MDS and changes in MDS over time on BP outcomes. While higher baseline adherence to the MD was associated with sustained improvements in BP at follow-up 2, changes in MDS during the study period showed inconsistent effects on BP regulation. This discrepancy could be attributed to the time-dependent nature of dietary effects, where long-term adherence to high-quality diets exerts more pronounced benefits than short-term dietary changes. Furthermore, these results highlight the potential role of dietary stability in cardiovascular health, suggesting that consistent prolonged adherence to a healthy dietary pattern such as the MD may be more beneficial than sporadic improvements [41]. Future studies should investigate whether specific subgroups experience more substantial changes in MDS over time, as the modest decline in MDS observed in this study may not have been sufficient to detect more pronounced changes in BP. More frequent dietary assessments and advanced modeling techniques could help capture dynamic dietary patterns and their temporal associations with BP regulation.

Expanding on the biological mechanisms, the observed associations between MDS and BP may be mediated by several key components of the MD, including high intakes of bioactive secondary plant metabolites such as polyphenols [42], monounsaturated fatty acids (MUFAs) [43], and omega-3 fatty acids [44]. These bioactive compounds have been shown to improve endothelial function, reduce arterial stiffness, and attenuate inflammation factors that play critical roles in BP regulation [45], either via direct antioxidant, i.e., quenching mechanisms [46, 47] or by acting on transcription factors such as Nrf-2 and NF-ĸB [46, 48]. Furthermore, polyphenols found in fruits, vegetables, and olive oil are known to enhance nitric oxide bioavailability, leading to vasodilation and lower vascular resistance [49, 50]. Similarly, omega-3 fatty acids from fish and nuts have been shown to modulate inflammatory pathways and reduce oxidative stress by promoting the formation of resolvins and protectins [51], thereby contributing to improved vascular health [51]. Investigating these mechanisms in greater detail could provide valuable insights into how specific dietary components in the MD interact to produce cardioprotective effects, offering a pathway for targeted dietary recommendations and potential nutraceutical development.

Our findings also raise important considerations regarding the role of age and sex as potential modifiers of the association between diet quality and BP outcomes. Although we adjusted for these variables in our analyses, the differential physiological and hormonal changes associated with aging and sex-specific factors, such as menopausal status in women, may influence the observed effects of the MD. Previous studies have suggested that the cardioprotective effects of the MD may be more pronounced in older adults, potentially due to their higher baseline risk of hypertension and other cardiometabolic disorders [52–54]. Similarly, sex-specific responses to dietary interventions, such as lipid metabolism or vascular function differences, could account for outcome variations [55]. Future research should explore these interactions in greater depth, employing stratified analyses or interaction models to identify subgroups that may benefit most from dietary interventions.

Our findings underscore the importance of promoting healthy dietary patterns, such as the MD, as part of public health initiatives to prevent hypertension and other cardiometabolic diseases, particularly in older populations. The observed long-term benefits suggest that early dietary interventions could have lasting effects on BP regulation and, thus, overall cardiovascular health. Hypertension is also strongly associated with multimorbidity, often coexisting with other chronic conditions such as diabetes [56], obesity [57], and kidney disease [58, 59]. When present together, these conditions create a complex interplay that significantly exacerbates health outcomes [60]. For instance, the combination of hypertension and diabetes accelerates the development of cardiovascular complications [61], while obesity further amplifies the strain on the cardiovascular system [62]. This co-occurrence of multiple chronic conditions often leads to greater healthcare utilization, decreased quality of life, and an increased risk of premature mortality [63]. Managing these interconnected conditions requires a comprehensive approach that addresses the underlying risk factors, including diet, and emphasizes early intervention, lifestyle changes, coordinated care, and the adoption of dietary patterns such as the MD.

Strengths of our study include the large number of participants and the nationwide character of the study, in addition to its longitudinal nature. Furthermore, using various statistical models, e.g., LMMs, LRMs, and LCSMs, allowed us to adjust for important confounders such as age, sex, physical activity, and dwelling type, which may influence BPM outcomes. Considering these factors helped ensure that our findings were robust and not driven by other external variables. For example, dwelling type may reflect socioeconomic factors that could impact both diet and health [64], while physical activity is a well-known modulator of cardiovascular health [65]. Future studies could further explore the role of these confounders, particularly in more diverse subgroups, to refine our understanding of how dietary patterns such as the MD interact with broader lifestyle factors in influencing BP.

Our study has several limitations. First, the observational nature of the analysis limits the ability to establish causality. While the longitudinal design strengthens the evidence for temporal association, unmeasured/uncontrolled confounders may still influence the results. Additionally, the reliance on self-reported dietary data introduces potential biases, such as recall bias or inaccuracies in the estimation of MDS. In addition, while blood pressure was selected as the primary outcome due to its clinical relevance and data availability, we acknowledge that it does not fully capture the complexity of cardiometabolic health; future analyses will incorporate additional biomarkers such as blood lipids and fasting glucose to provide a more comprehensive assessment. Future research could benefit from more robust dietary assessments, such as biomarkers of food intake [66], to further validate these findings. Moreover, exploring the underlying mechanisms linking diet and BP through inflammatory or oxidative stress pathways would provide valuable insights into how diet modulates cardiovascular risk. Long-term randomized controlled trials are also needed to confirm the causal association observed in observational studies and evaluate dietary interventions’ effectiveness in reducing BP and improving cardiometabolic health outcomes in diverse populations.

## Conclusion

Our study provides compelling evidence for the long-term benefits of healthy dietary patterns, particularly the MD, on BP regulation and cardiovascular health. The findings demonstrate that adherence to the MD, characterized by a high intake of fruits, vegetables, whole grains, legumes, and healthy fats, was associated with significant reductions in BP over time in older adults. This underscores the potential of diet as a modifiable risk factor for preventing hypertension and other cardiometabolic diseases, offering a practical and sustainable approach to managing public health issues.

To build on these findings, future studies should employ randomized controlled trials to confirm the causal association observed in this cohort and explore the biological mechanisms that mediate the effects of diet on cardiovascular health. Furthermore, larger, more diverse populations and the use of advanced dietary biomarkers of food intake could provide more substantial evidence for the generalizability and robustness of these findings. Ultimately, dietary interventions that promote long-term health and prevent the onset of hypertension could play a critical role in mitigating the growing burden of cardiovascular disease, particularly as global populations age.

## Data Availability

Data are available from the Canadian Longitudinal Study on Aging (www.clsa-elcv.ca) for researchers who meet the criteria for access to de-identified CLSA data.

## Declarations

### Disclaimer

The opinions expressed in this manuscript are the author’s own and do not reflect the views of the Canadian Longitudinal Study on Aging.

## References

[1] Rojas-Montesino E, Méndez D, Espinosa-Parrilla Y, Fuentes E, Palomo I (2022) Analysis of scientometric indicators in publications associated with healthy aging in the world, period 2011–2020. Int J Environ Res Public Health 19(15):8988 35897359 10.3390/ijerph19158988 PMC9329745

[2] Nations U (2024) Revision of World Population Prospects. In: Department of Economic and Social Affairs PD, editor.

[3] Chowdhury SR, Chandra Das D, Sunna TC, Beyene J, Hossain A (2023) Global and regional prevalence of Multimorbidity in the adult population in community settings: a systematic review and meta-analysis. EClinicalMedicine 57:101860. 10.1016/j.eclinm.2023.101860 36864977 10.1016/j.eclinm.2023.101860 PMC9971315

[4] WHO: Global Health Estimates (2024): Life expectancy and leading causes of death and disability. https://www.who.int/data/gho/data/themes/mortality-and-global-health-estimates Accessed 2024.

[5] Organization WH (2019) Global Burden of Disease Collaborative Network, Global Burden of Disease Study 2019 (GBD 2019) Results

[6] Saeedi P, Petersohn I, Salpea P, Malanda B, Karuranga S, Unwin N et al (2019) Global and regional diabetes prevalence estimates for 2019 and projections for 2030 and 2045: results from the international diabetes federation diabetes atlas, 9(th) edition. Diabetes Res Clin Pract 157:107843. 10.1016/j.diabres.2019.107843 31518657 10.1016/j.diabres.2019.107843

[7] Ong KL, Stafford LK, McLaughlin SA, Boyko EJ, Vollset SE, Smith AE et al (2023) Global, regional, and National burden of diabetes from 1990 to 2021, with projections of prevalence to 2050: a systematic analysis for the global burden of disease study 2021. Lancet 402(10397):203–234. 10.1016/S0140-6736(23)01301-6 37356446 10.1016/S0140-6736(23)01301-6 PMC10364581

[8] Shi H, Xia Y, Cheng Y, Liang P, Cheng M, Zhang B et al (2024) Global burden of ischemic heart disease from 2022 to 2050: projections of incidence, prevalence, deaths, and Disability-Adjusted life years. Eur Heart J Qual Care Clin Outcomes. 10.1093/ehjqcco/qcae049 38918062 10.1093/ehjqcco/qcae049

[9] Harris E (2024) Heart disease prevalence, medical costs will soar in US by 2050. JAMA 332(4):273. 10.1001/jama.2024.11026 38941123 10.1001/jama.2024.11026

[10] Chong B, Jayabaskaran J, Jauhari SM, Chan SP, Goh R, Kueh MTW et al (2024) Global burden of cardiovascular diseases: projections from 2025 to 2050. Eur J Prev Cardiol. 10.1093/eurjpc/zwae281 10.1093/eurjpc/zwae281 39270739

[11] Rony MKK, Parvin MR, Wahiduzzaman M, Akter K, Ullah M (2024) Challenges and advancements in the health-related quality of life of older people. Adv Public Health 2024(1):8839631

[12] Ruiz-Castell M, Kandala N-B, Kuemmerle A, Schritz A, Barré J, Delagardelle C et al (2016) Hypertension burden in Luxembourg: individual risk factors and geographic variations, 2013 to 2015 European health examination survey. Medicine 95(36):e4758. 10.1097/md.0000000000004758 27603374 10.1097/MD.0000000000004758 PMC5023897

[13] Seshadri S, Wolf PA, Beiser A, Vasan RS, Wilson PW, Kase CS et al (2001) Elevated midlife blood pressure increases stroke risk in elderly persons: the Framingham study. Arch Intern Med 161(19):2343–2350 11606150 10.1001/archinte.161.19.2343

[14] Fuchs FD, Whelton PK (2020) High blood pressure and cardiovascular disease. Hypertension 75(2):285–292 31865786 10.1161/HYPERTENSIONAHA.119.14240 PMC10243231

[15] Zhang K, Ma Y, Luo Y, Song Y, Xiong G, Ma Y et al (2023) Metabolic diseases and healthy aging: identifying environmental and behavioral risk factors and promoting public health. Front Public Health 11:1253506 37900047 10.3389/fpubh.2023.1253506 PMC10603303

[16] Lee PH, Wong FK (2015) The association between time spent in sedentary behaviors and blood pressure: a systematic review and meta-analysis. Sports Med 45:867–880 25749843 10.1007/s40279-015-0322-y

[17] Kumar V, Huligowda LKD, Umesh M, Chakraborty P, Thazeem B, Singh AP (2025) Environmental pollutants as emerging concerns for cardiac diseases: A review on their impacts on cardiac health. Biomedicines 13(1):241 39857824 10.3390/biomedicines13010241 PMC11759859

[18] D’Souza J, Weuve J, Brook RD, Evans DA, Kaufman JD, Adar SD (2021) Long-term exposures to urban noise and blood pressure levels and control among older adults. Hypertension 78(6):1801–1808 34689591 10.1161/HYPERTENSIONAHA.121.17708 PMC8585701

[19] Dong OM (2018) Excessive dietary sodium intake and elevated blood pressure: a review of current prevention and management strategies and the emerging role of pharmaconutrigenetics. BMJ Nutr Prev Health 1(1):7 33235949 10.1136/bmjnph-2018-000004 PMC7678480

[20] Abrignani V, Salvo A, Pacinella G, Tuttolomondo A (2024) The mediterranean diet, its Microbiome connections, and cardiovascular health: A narrative review. Int J Mol Sci 25(9). 10.3390/ijms25094942 10.3390/ijms25094942 PMC11084172 38732161

[21] Georgoulis M, Damigou E, Derdelakou E, Kosti RI, Chrysohoou C, Barkas F et al (2024) Adherence to the mediterranean diet and 20-year incidence of hypertension: the ATTICA prospective epidemiological study (2002–2022). Eur J Clin Nutr 78(7):630–638. 10.1038/s41430-024-01440-w 38605190 10.1038/s41430-024-01440-w

[22] Sarsangi P, Salehi-Abargouei A, Ebrahimpour-Koujan S, Esmaillzadeh A (2022) Association between adherence to the mediterranean diet and risk of type 2 diabetes: an updated systematic review and Dose-Response Meta-Analysis of prospective cohort studies. Adv Nutr 13(5):1787–1798. 10.1093/advances/nmac046 35472102 10.1093/advances/nmac046 PMC9526848

[23] Sebastian SA, Padda I, Johal G (2024) Long-term impact of mediterranean diet on cardiovascular disease prevention: A systematic review and meta-analysis of randomized controlled trials. Curr Probl Cardiol 49(5):102509. 10.1016/j.cpcardiol.2024.102509 38431146 10.1016/j.cpcardiol.2024.102509

[24] Elsner RJF (2002) Changes in eating behavior during the aging process. Eat Behav 3(1):15–43. 10.1016/S1471-0153(01)00041-1 15001018 10.1016/s1471-0153(01)00041-1

[25] Fekete M, Szarvas Z, Fazekas-Pongor V, Feher A, Csipo T, Forrai J et al (2022) Nutrition strategies promoting healthy aging: from improvement of cardiovascular and brain health to prevention of Age-Associated diseases. Nutrients 15(1). 10.3390/nu15010047 10.3390/nu15010047 PMC9824801 36615705

[26] Kassis A, Fichot MC, Horcajada MN, Horstman AMH, Duncan P, Bergonzelli G et al (2022) Nutritional and lifestyle management of the aging journey: A narrative review. Front Nutr 9:1087505. 10.3389/fnut.2022.1087505 36761987 10.3389/fnut.2022.1087505 PMC9903079

[27] Raina PS, Wolfson C, Kirkland SA, Griffith LE, Oremus M, Patterson C et al (2009) The Canadian longitudinal study on aging (CLSA). Can J Aging / La Revue Canadienne Du Vieillissement 28(3):221–229. 10.1017/S0714980809990055 10.1017/S0714980809990055 19860977

[28] Raina P, Wolfson C, Kirkland S, Griffith LE, Balion C, Cossette B et al (2019) Cohort Profile: The Canadian Longitudinal Study on Aging (CLSA). International Journal of Epidemiology.;48(6):1752-3j. 10.1093/ije/dyz173 10.1093/ije/dyz173 PMC6929533 31633757

[29] Shatenstein B, Payette H (2015) Evaluation of the relative validity of the short diet questionnaire for assessing usual consumption frequencies of selected nutrients and foods. Nutrients 7(8):6362–6374 26247965 10.3390/nu7085282 PMC4555121

[30] Aoun C, Papazian T, Helou K, El Osta N, Khabbaz LR (2019) Comparison of five international indices of adherence to the mediterranean diet among healthy adults: similarities and differences. Nutr Res Pract 13(4):333–343 31388410 10.4162/nrp.2019.13.4.333 PMC6669066

[31] DeMers D, Wachs D (2019) Physiology, mean arterial pressure 30855814

[32] Henry JB, Miller MC, Kelly KC, Champney D (2002) Mean arterial pressure (MAP): an alternative and preferable measurement to systolic blood pressure (SBP) in patients for hypotension detection during hemapheresis. J Clin Apheresis: Official J Am Soc Apheresis 17(2):55–64 10.1002/jca.10022 12210707

[33] Sesso HD, Stampfer MJ, Rosner B, Hennekens CH, Gaziano JM, Manson JE, Glynn RJ (2000) Systolic and diastolic blood pressure, pulse pressure, and mean arterial pressure as predictors of cardiovascular disease risk in men. Hypertension 36(5):801–807 11082146 10.1161/01.hyp.36.5.801

[34] Vahid F, Hoge A, Hébert JR, Bohn T, Alkerwi Aa, Noppe S et al (2023) Association of diet quality indices with serum and metabolic biomarkers in participants of the ORISCAV-LUX-2 study. Eur J Nutr 62(5):2063–2085. 10.1007/s00394-023-03095-y 36917281 10.1007/s00394-023-03095-y PMC10349755

[35] Vahid F, Chiriboga D, Bohn T, Hébert JR (2022) Chapter 8 - Diet, inflammation, and cardiovascular disease. In: Hébert JR, Hofseth LJ (eds) Diet, inflammation, and health. Academic, pp 367–472

[36] Al Kudsee K, Vahid F, Bohn T (2022) High adherence to the mediterranean diet and alternative healthy eating index are associated with reduced odds of metabolic syndrome and its components in participants of the ORISCAV-LUX2 study. Front Nutr 9:1087985 36583217 10.3389/fnut.2022.1087985 PMC9793091

[37] De Pergola G, D’Alessandro A (2018) Influence of mediterranean diet on blood pressure. Nutrients 10(11):1700 30405063 10.3390/nu10111700 PMC6266047

[38] Davis CR, Hodgson JM, Woodman R, Bryan J, Wilson C, Murphy KJ (2017) A mediterranean diet lowers blood pressure and improves endothelial function: results from the medley randomized intervention trial. Am J Clin Nutr 105(6):1305–1313 28424187 10.3945/ajcn.116.146803

[39] Papadaki A, Nolen-Doerr E, Mantzoros CS (2020) The effect of the mediterranean diet on metabolic health: A systematic review and Meta-Analysis of controlled trials in adults. Nutrients 12(11). 10.3390/nu12113342 10.3390/nu12113342 PMC7692768 33143083

[40] Toledo E, Hu FB, Estruch R, Buil-Cosiales P, Corella D, Salas-Salvadó J et al (2013) Effect of the mediterranean diet on blood pressure in the PREDIMED trial: results from a randomized controlled trial. BMC Med 11(1):207. 10.1186/1741-7015-11-207 24050803 10.1186/1741-7015-11-207 PMC3849640

[41] Rosato V, Temple NJ, La Vecchia C, Castellan G, Tavani A, Guercio V (2019) Mediterranean diet and cardiovascular disease: a systematic review and meta-analysis of observational studies. Eur J Nutr 58(1):173–191. 10.1007/s00394-017-1582-0 29177567 10.1007/s00394-017-1582-0

[42] Godos J, Vitale M, Micek A, Ray S, Martini D, Del Rio D et al (2019) Dietary polyphenol intake, blood pressure, and hypertension: a systematic review and meta-analysis of observational studies. Antioxidants 8(6):152 31159186 10.3390/antiox8060152 PMC6616647

[43] Miura K, Stamler J, Brown IJ, Ueshima H, Nakagawa H, Sakurai M et al (2013) Relationship of dietary monounsaturated fatty acids to blood pressure: the international study of macro/micronutrients and blood pressure. J Hypertens 31(6):1144–1150 23572200 10.1097/HJH.0b013e3283604016 PMC4109685

[44] Wang H, Li Q, Zhu Y, Zhang X (2021) Omega-3 polyunsaturated fatty acids: versatile roles in blood pressure regulation. Antioxid Redox Signal 34(10):800–810 32349540 10.1089/ars.2020.8108

[45] Firdous SM, Pal S (2025) Antioxidants in Cardiovascular Disease: Molecular Interaction and Therapeutic Implications. Antioxidants: Nature’s Defense Against Disease.:127– 51

[46] Caminiti R, Carresi C, Mollace R, Macrì R, Scarano F, Oppedisano F et al (2024) The potential effect of natural antioxidants on endothelial dysfunction associated with arterial hypertension. Front Cardiovasc Med 11:1345218 38370153 10.3389/fcvm.2024.1345218 PMC10869541

[47] Grosso G, Godos J, Currenti W, Micek A, Falzone L, Libra M et al (2022) The effect of dietary polyphenols on vascular health and hypertension: current evidence and mechanisms of action. Nutrients 14(3):545 35276904 10.3390/nu14030545 PMC8840535

[48] Gabriele M, Pucci L (2017) Diet bioactive compounds: implications for oxidative stress and inflammation in the vascular system. Endocrine, metabolic & immune Disorders-Drug targets (Formerly current drug targets-Immune. Endocr Metabolic Disorders) 17(4):264–275 10.2174/1871530317666170921142055 28933306

[49] Yamagata K (2019) Polyphenols regulate endothelial functions and reduce the risk of cardiovascular disease. Curr Pharm Design 25(22):2443–2458 10.2174/1381612825666190722100504 31333108

[50] Monsalve B, Concha-Meyer A, Palomo I, Fuentes E (2017) Mechanisms of endothelial protection by natural bioactive compounds from fruit and vegetables. An Acad Bras Cienc 89:615–633 28538813 10.1590/0001-3765201720160509

[51] Oppedisano F, Macrì R, Gliozzi M, Musolino V, Carresi C, Maiuolo J et al (2020) The anti-inflammatory and antioxidant properties of n-3 PUFAs: their role in cardiovascular protection. Biomedicines 8(9):306 32854210 10.3390/biomedicines8090306 PMC7554783

[52] Pant A, Chew DP, Mamas MA, Zaman S (2024) Cardiovascular disease and the mediterranean diet: insights into Sex-Specific responses. Nutrients 16(4). 10.3390/nu16040570 10.3390/nu16040570 PMC10893368 38398894

[53] Dontas AS, Zerefos NS, Panagiotakos DB, Vlachou C, Valis DA (2007) Mediterranean diet and prevention of coronary heart disease in the elderly. Clin Interv Aging 2(1):109–115. 10.2147/ciia.2007.2.1.109 18044083 10.2147/ciia.2007.2.1.109 PMC2684076

[54] Furbatto M, Lelli D, Antonelli Incalzi R, Pedone C (2024) Mediterranean diet in older adults: cardiovascular outcomes and mortality from observational and interventional Studies—A systematic review and Meta-Analysis. Nutrients. 10.3390/nu16223947 39599734 10.3390/nu16223947 PMC11597443

[55] Andrews RR, Anderson KR, Fry JL (2024) Sex-specific variation in metabolic responses to diet. Nutrients 16(17):2921 39275236 10.3390/nu16172921 PMC11397081

[56] Berbari AE, Daouk NA, Nasr EM (2023) Coexistence of diabetes mellitus and hypertension. Blood pressure disorders in diabetes mellitus. Springer, pp 3–17

[57] Dorresteijn J, Visseren F, Spiering W (2012) Mechanisms linking obesity to hypertension. Obes Rev 13(1):17–26 21831233 10.1111/j.1467-789X.2011.00914.x

[58] Tedla F, Brar A, Browne R, Brown C (2011) Hypertension in chronic kidney disease: navigating the evidence. Int J Hypertens 2011(1):132405 21747971 10.4061/2011/132405 PMC3124254

[59] Hall ME, do Carmo JM, da Silva AA, Juncos LA, Wang Z, Hall JE (2014) Obesity, hypertension, and chronic kidney disease. Int J Nephrol Renovascular Disease.:75–88 10.2147/IJNRD.S39739 PMC3933708 24600241

[60] Hezam AAM, Shaghdar HBM, Chen L (2024) The connection between hypertension and diabetes and their role in heart and kidney disease development. J Res Med Sciences: Official J Isfahan Univ Med Sci. 29 10.4103/jrms.jrms_470_23 PMC11162087 38855561

[61] Petrie JR, Guzik TJ, Touyz RM (2018) Diabetes, hypertension, and cardiovascular disease: clinical insights and vascular mechanisms. Can J Cardiol 34(5):575–584 29459239 10.1016/j.cjca.2017.12.005 PMC5953551

[62] Mwende Wairimu G Cardiovascular Risk in Patients with Coexisting Diabetes and Hypertension: A Comprehensive Review

[63] van der Zee-Neuen A, Putrik P, Ramiro S, Keszei A, de Bie R, Chorus A, Boonen A (2016) Impact of chronic diseases and Multimorbidity on health and health care costs: the additional role of musculoskeletal disorders. Arthritis Care Res 68(12):1823–1831 10.1002/acr.22913 27111195

[64] Vidal DG, Oliveira GM, Pontes M, Maia RL, Ferraz MP (2022) Chapter 6 - The influence of social and economic environment on health. In: Prata JC, Ribeiro AI, Rocha-Santos T (eds) One health. Academic, pp 205–229

[65] Sanchis-Gomar F, Lavie CJ, Marín J, Perez-Quilis C, Eijsvogels TM, O’Keefe JH et al (2022) Exercise effects on cardiovascular disease: from basic aspects to clinical evidence. Cardiovascular Res 118(10):2253–2266 10.1093/cvr/cvab272 34478520

[66] Cifuentes M, Vahid F, Devaux Y, Bohn T (2024) Biomarkers of food intake and their relevance to metabolic syndrome. Food Funct 15(14):7271–7304. 10.1039/d4fo00721b. 10.1039/d4fo00721b 38904169

